# Influenza vaccines may protect against cardiovascular diseases: The evidence is mounting and should be known by the Canadian public health community

**DOI:** 10.14745/ccdr.v49i10a04

**Published:** 2023-10-01

**Authors:** Philippe De Wals, Michaël Desjardins

**Affiliations:** 1 Department of Social and Preventive Medicine, Laval University, Québec City, QC; 2 Institut national de Santé publique du Québec, Québec City, QC; 3 Division of Infectious Diseases, Centre hospitalier de l’Université de Montréal, Montréal, QC

**Keywords:** influenza infection, influenza vaccine, cardiovascular disease, pneumonia

## Abstract

Evidence on the protective effect of influenza vaccines to prevent cardiovascular disease (CVD) is mounting. We identified 28 systematic reviews/meta-analyses on the effect of influenza vaccines on CVD using different research questions, data sources, selection criteria and outcomes. Most results leaned towards a protective effect. Results of recently published experimental and observational studies not included in these reviews were going in the same direction. The evidence is very robust for cardiovascular deaths and nonfatal myocardial infarction in high-risk individuals, but lower for heart failure, arrhythmia, and stroke and also for all outcomes in low-risk adults. There is also limited evidence for pneumococcal polysaccharide vaccines and evidence has to be collected from ongoing trials on respiratory syncytial virus vaccines. Up to now, this effect has not been considered in economic evaluations of influenza vaccines and its inclusion may change CVD results markedly. This effect is not mentioned in the Canadian Immunization Guide and not known by a majority of vaccinators. The objective of this short commentary is to alert the Canadian public health community and to provide information that could be used at the field level to promote the usefulness of influenza vaccines.

## Introduction

Evidence on the protective effect of influenza vaccines to prevent cardiovascular disease (CVD) is mounting. Recognition of this effect could modify results of economic evaluations markedly and also the way they are promoted. Influenza infections in adults are associated with an increased risk of adverse cardiovascular events, including sudden death, myocardial infarction, heart failure, cardiac arrhythmia, and stroke ((1,2)). One particularly interesting study was conducted in Ontario, showing that the frequency of hospital admissions for acute myocardial infarction was much higher during the seven days after a laboratory-confirmed influenza infection than during a control period (20.0 admissions per week vs. 3.3 admissions per week; rate ratio: 6.05, 95% CI: 3.86–9.50) ((3)). In this study, respiratory specimens that tested for influenza infection using high-specificity methods were submitted from physician offices, emergency departments, hospitals, long-term care facilities, and public health departments as part of routine clinical care, outbreak investigations, or research, meaning a wide array of clinical presentations and infection severity. Hospitalizations for acute myocardial infarction were obtained from the Discharge Abstract Database of the Canadian Institute for Health Information. The self-controlled case-series method was applied in which only individuals who experienced an event of interest are included and are acting as their own control (risk vs. control period), meaning that time invariant confounders such as comorbidities are eliminated ((4)). In an ecological analysis of vital registration data in ten countries, the fraction of ischaemic heart deaths attributable to influenza was estimated at 3.9%, ranging from less than 1% to 10% according to country and year ((5)).

Several biological mechanisms have been proposed to explain how an infection could trigger a CVD: 1) the induction of pro-inflammatory changes in the cellular composition of atherosclerotic lesions, 2) the induction of a persistent pro-coagulant state, including platelet activation, 3) the increased metabolic needs of peripheral tissues and organs compromising arterial perfusion, and 4) the infection and inflammation of myocardial cells disturbing the cardiac function ((6,7)).

## Protective effect of vaccines

The protective role of influenza vaccination on CVD death was first raised by Meyers in a review of one clinical trial and three epidemiological studies published in 2003 ((8)). The first Cochrane review on the association between influenza vaccination and cardiovascular risk reduction was published in 2008 and updated in 2015 ((9,10)). We conducted a PubMed search that identified 28 systematic reviews with or without meta-analysis on the protective effect of influenza vaccination on CVD, seven of which were published in 2022–2023 (see details in the **Supplemental material**). As seen in **Table 1**, these reviews focused on different questions, and used different data sources, selection criteria, and outcomes. Most results leaned towards a protective effect as shown in the most comprehensive review covering 33 studies. Out of 52 comparisons reported in the manuscript, 40 showed a statistically significant reduction in risk, 11 showed a non-statistically significant reduction in risk, and in only one comparison, a non-statistically significant increased risk of 2% was observed for stroke (11). The evidence is particularly strong for the occurrence of cardiac events in high-risk patients, which is supported by results from four randomized clinical trials (two of high quality and two of low quality) showing a 45% decrease risk (95% CI: 25%–59%) of major adverse cardiovascular events (cardiovascular death or hospitalization for myocardial infarction, unstable angina, stroke, heart failure, or urgent coronary revascularization) among participants who had a history of coronary disease and within 12 months of follow-up ((12)). It should be noted that there is much overlap between risk factors for complication of influenza infection and cardiovascular disease in adults, with one exception: hypertension, a frequent condition among the adult population of Canada (24%) but not included in the Canadian Immunization Guide list of conditions for which influenza vaccination is particularly recommended (13,14).

**Table 1:** Main characteristics of meta-analyses published in 2021–2023, and pertaining to the potential effect of influenza vaccines on cardiovascular disease in adults

Reference	Objectives	Number of studies included	Main results^a^
Diaz-Arocutipa *et al.,* 2022 ((15))	To evaluate the effect of the influenza vaccine on cardiovascular outcomes in patients with coronary artery disease.	5 RCTs involving 4,211 patients	Influenza vaccine significantly reduced the risk of major adverse cardiovascular event (RR: 0.63, 95% CI: 0.51–0.77), all-cause mortality (RR: 0.58, 95% CI: 0.40–0.84) and cardiovascular mortality (RR: 0.53, 95% CI: 0.38–0.74). Reduction in the risk of myocardial infarction was not statistically significant (RR: 0.69, 95% CI: 0.47–1.02).
Maniar *et al.,* 2022 ((16))	Updated meta-analysis including all RCTs that evaluated influenza vaccine and its association with cardiovascular outcomes.	8 RCTs with a total of 14,420 patients	Influenza vaccine, as compared with control/placebo, was associated with significantly lower risk of major adverse cardiovascular events at the follow-up (RR: 0.75, 95% CI: 0.57–0.97).
Gupta *et al.,* 2022 ((17))	Systematic review and meta-analysis addressing whether vaccination against influenza reduces adverse vascular events and mortality in heart failure patients.	7 non-randomized studies with a total of 247,842 patients	The risk of all-cause mortality is significantly reduced within 12 months of a heart failure patient receiving the influenza vaccine (RR: 0.75, 95% CI: 0.71–0.79); very low certainty of evidence. The risk of cardiovascular-related mortality was significantly reduced (RR: 0.77, 95% CI: 0.73–0.81); low certainty of evidence. The pooled risk of all-cause hospitalization was higher among vaccinated heart failure patients (RR: 1.24, 95% CI: 1.13–1.35), based on two studies; very low certainty of evidence.
Jaiswal *et al.,* 2022 ((18))	To estimate the effect of influenza vaccination on cardiovascular and cerebrovascular outcomes among patients with established CVD.	5 RCTs and 13 observational studies, with a total of 22,532,165 patients were included	At a mean follow-up of 1.5 years, the vaccinated group was associated with a lower risk of all-cause mortality (HR: 0.71, 95% CI: 0.63–0.80), major adverse cardiovascular event (HR: 0.83, 95% CI: 0.72–0.96), cardiovascular mortality (HR: 0.78, 95% CI: 0.68–0.90), and MI (HR: 0.82, 95% CI: 0.74–0.92). The incidence of stroke (HR: 1.03, 95% CI: 0.92–1.06) and heart failure (HR: 0.74, 95% CI: 0.51–1.08) did not differ between the two groups.
Behrouzi *et al.,* 2022 ((12))	To evaluate if seasonal influenza vaccination is associated with a lower risk of fatal and non-fatal cardiovascular events.	6 published RCTs comprising a total of 9,001 participants	Influenza vaccination was associated with a lower risk of composite cardiovascular events (3.6% vs. 5.4%; RR: 0.66, 95% CI: 0.53–0.83). Protection was demonstrated among patients with recent acute coronary syndrome (RR: 0.55, 95% CI: 0.41–0.75) but not in those without cardiac disease history (RR: 1.00, 95% CI: 0.68–1.47).
Tavabe *et al.,* 2023 ((19))	To identify studies that investigated the potential effects of the influenza vaccine on arrhythmia risk.	1 RCT with 2,532 patients and 6 observational studies with 3,167,445 patients were included	One RCT demonstrated a non-significant benefit against arrhythmia: (OR: 0.43, 95% CI: 0.11–1.64) in patients after myocardial infarction or those with high-risk stable coronary heart disease. A meta-analysis based on observational studies showed that vaccination was associated with a significantly lower risk of arrhythmia (OR: 0.82, 95% CI: 0.70–0.97).
Liu *et al.,* 2023 ((20))	To investigate the relationship between receiving the flu vaccine with stroke and its hospitalization in the elderly.	14 observational studies were included for a total of 3,198,646 participants	Summary OR of occurrence and hospitalization of stroke compared to the unvaccinated group in vaccine recipients was 0.84 (95% CI: 0.78–0.90).
Addario *et al.,* 2023 ((11))	To summarize the impact of vaccination against influenza, shingles, and pneumococcus on the risk of cardiovascular events in persons 65 years of age and older.	A total of 33 studies pertaining to influenza vaccination were analyzed	Out of 52 comparisons reported in the manuscript, 40 showed a statistically significant reduction in risk, 11 a non-statistically significant reduction in risk, and, in only one comparison, a non-statistically significant increased risk of 2% was observed. Also, repeated influenza vaccination showed a consistent and dose-dependent protective effect against acute coronary syndromes and stroke.
Gupta *et al.,* 2023 2023 ((21))	To provide evidence regarding the protective effects of influenza vaccination in patients with cardiovascular disease.	15 studies with a total of 745,001 patients were included in the analysis, including 6 RCTs, 7 retrospective cohort studies, and 2 case-control studies	Lower rates of all-cause mortality (OR: 0.74, 95% CI: 0.64–0.86), cardiovascular death (OR: 0.73, 95% CI: 0.59–0.92), and stroke (OR: 0.71, 95% CI: 0.57–0.89) were observed. There was no significant statistical difference in rates of myocardial infarction (OR: 0.91, 95% CI: 0.69–1.21) or heart failure hospitalizations (OR: 1.06, 95% CI: 0.85–1.31).
Modin *et al.,* 2023 ((22))	Meta-analysis of RCTs to assess the effect of influenza vaccination on the incidence of cardiovascular events in patients with ischaemic heart disease or heart failure.	5 peer-reviewed RCTs and 1 non-peer-reviewed RCT, for a total of 9,340 patients, were included. The primary endpoint was a composite of cardiovascular death, acute coronary syndrome, stent thrombosis or coronary revascularization, stroke or heart failure hospitalization	Influenza vaccination was associated with a reduced incidence of the primary composite endpoint (random effects HR: 0.74, 95% CI: 0.63–0.88, *p*<0.001, I2=52%), cardiovascular death (rHR: 0.63, 95% CI: 0.42–0.95, *p*=0.028, I2=58%) and all-cause death (rHR: 0.72, 95% CI: 0.54–0.95, *p*=0.0227, I2=52%).

Abbreviations: CI, confidence interval; CVD, cardiovascular disease; HR, hazard ratio; MI, myocardial infarction; OR, odds ratio; RCT, randomized controlled trial; rHR, randomized hazard ratio; RR, risk ratio

^a^ Vaccine efficacy/protection=(1 RR/HR/OR) expressed as a percentage

Results of recently published studies not included in these reviews provide additional evidence. In a multicentric randomized clinical trial on the effect of inactivated influenza vaccine among patients with chronic heart failure, vaccination did not significantly reduce the first primary composite outcome (cardiovascular death, nonfatal myocardial infarction, or non-fatal stroke) during the entire three-year trial period, whereas vaccination reduced community-acquired pneumonia by 42% (95% CI: 20%–58%). During peak influenza circulation periods, however, a statistically significant protective effect of 18% (95% CI: 1%–32%) was observed against the composite CVD outcome ((23)). Administrative data from the Alberta Health Care Insurance Plan were analyzed to assess the risk of stroke event comprising acute ischaemic stroke, intracerebral haemorrhage, subarachnoid haemorrhage, and transient ischaemic attack, following influenza vaccination during the period 2009–2018. Adjusted for demographics and comorbidities, recent influenza vaccination provided a statistically significant protection of 22% (95% CI: 21%–24%) ((24)).

## Economic analyses

Cardiovascular outcomes are rarely incorporated into economic evaluations of influenza vaccines. Hospitalizations for these outcomes have been specifically included in a few piggyback economic evaluations of high-dose inactivated influenza vaccine trials reported in a systematic review, which concluded that reduced cardiorespiratory complications were an important driver of the economic benefits of vaccination (25). Pneumonia could directly result in death or be a contributing cause of a more distant fatal outcome, but permanent sequelae are not frequent ((26)). The long-term consequences of adverse cardiovascular outcomes are much more severe, for stroke especially ((27)). In a meta-analysis of the cost effectiveness of influenza vaccination in the elderly in high-income countries, the conclusion was that incremental cost-effectiveness ratios in a societal perspective were favourable regardless of the types of vaccines ((28)). This is not necessarily the case when a health-system perspective is adopted. In Québec, an economic evaluation of the standard dose-inactivated influenza vaccination concluded that it was not cost-effective among the groups with chronic conditions aged 5–64 years and for healthy individuals of any age, approaching the cost-effectiveness threshold ($45,000/QALY, quality-adjusted life year, corresponding to the per capita gross domestic product in Canada in 2015) for healthy individuals aged 75 years and over ((29)). Accordingly, it was proposed to withdraw healthy adults aged 60–74 years from the list of groups at high-risk for influenza-associated hospitalization and death who had free access to vaccination.

The inclusion of cardiovascular outcomes in the base-case scenario (outcomes with high-level evidence) and in sensitivity analyses (outcomes with moderate-level evidence) of economic evaluation of vaccines targeting respiratory infections could change the results of economic evaluation markedly, especially for high-risk groups. This is especially important in the context of increasing availability of new-generation influenza vaccines having a higher purchase cost than older ones.

## Promotion of vaccination

Results from the 2021–2022 Seasonal Influenza Vaccination Coverage Survey showed that overall uptake in the adult population was 39%, reaching 71% among seniors 65 years of age and older but only 38% among adults aged 18–64 years with a chronic medical condition, well below the national coverage goals of 80% ((30)). In 2021, the American College of Cardiology and the World Heart Federation published a statement focusing on the effect of influenza vaccines on CVD ((4)). Although the importance of the CVD protection may be well known by cardiologists, it is certainly not the case in the Canadian public health network, as it is not mentioned in the most recent statement on seasonal influenza vaccination (2022–2023) of the National Advisory Committee on Immunization nor in the Canadian Immunization Guide (14).

In Denmark, a cluster-randomized trial was conducted during the 2022–2023 influenza season among about one million citizens aged 65 years and older ((31)). Households were randomly assigned to usual care, or were sent nine different short electronic letters, designed on the basis of different behavioural concepts. Compared with usual care, influenza vaccination rates were higher in the group that received an electronic letter that highlighted the potential cardiovascular benefits of vaccination (81.00% vs. 80.12%; difference 0.89% points [95% CI: 0.29–1.48]). Other letters that did not highlight the potential cardiovascular benefits of vaccination (7 out of 9) were ineffective, except for the one that provided a reminder. Although the magnitude of the effect of this ultra-light intervention was modest, this is a “proof-of-concept” that elderly individuals are receptive to information about their risk of cardiovascular disease. More research is needed to assess the field impact of CVD messaging provided by healthcare providers including family physicians and pharmacists.

## Conclusion

The available evidence of a protective effect of influenza vaccines on CVD outcomes is sufficiently robust to include this effect in future economic evaluations. To mention this potential effect may change the perception of the population on the usefulness of influenza vaccines and increase vaccine uptake. Messages prepared by public health authorities and information provided to patients by vaccinators, including family physicians, nurses and pharmacists, should contain updated information on this issue. Severe acute respiratory syndrome coronavirus 2 (SARS-CoV-2), respiratory syncytial virus, and *Streptococcus pneumoniae* infections can also trigger adverse cardiovascular outcomes that may be prevented by vaccination ((3,32,33)). The evidence is robust for COVID vaccines but not for pneumococcal vaccines, due to the absence of high-quality studies. The evidence is still to be collected for the new respiratory syncytial virus vaccines for adults that will be marketed in the near future.

## Supplemental material

These documents can be accessed on the Supplemental material file.
